# Editorial: Newborn Screening for Inborn Errors of Metabolism: Is It Time for a Globalized Perspective Based on Genetic Screening?

**DOI:** 10.3389/fgene.2021.758142

**Published:** 2021-09-13

**Authors:** Mohamed A. Elmonem, Lambertus P. van den Heuvel

**Affiliations:** ^1^Department of Clinical and Chemical Pathology, Faculty of Medicine, Cairo University, Cairo, Egypt; ^2^Department of Pediatric Nephrology & Development and Regeneration, KU Leuven, Leuven, Belgium; ^3^Department of Pediatric Nephrology, Radboud University Medical Centre, Nijmegen, Netherlands

**Keywords:** newborn screening (NBS), inborn errors of metabolism (IEMs), next generation sequencing - NGS, tandem mass spectrometry, pathogenic variants, dried blood spots, clinical outcome

Inborn errors of metabolism (IEMs) are a large group of debilitating hereditary disorders, commonly manifesting during infancy and early childhood (Mak et al., 2013). They are categorized mainly according to the chemical nature of the characteristic metabolites accumulating in each disease. Major categories include aminoacidopathies, organic acid disorders, lysosomal storage disorders, fatty acid oxidation defects, and many others. Collectively, they constitute over 1,000 individual genetic disorders resulting in substantial societal and financial burdens overloading families, communities, and health care authorities worldwide (Péntek et al., 2016; Zeltner et al., 2017). These burdens are only expected to go higher under the current pandemic situation necessitating better planning for a better care of such disorders in the future (Elmonem et al., 2020).

Historically, the concept of newborn screening (NBS) was first applied to the amino acid metabolic disorder phenylketonuria using a simple bacterial inhibition assay to detect high phenylalanine concentrations in dried blood spots almost 60 years ago (Guthrie and Susi, 1963). Since then, the technologies used, and the metabolites discovered for the newborn screening of various IEMs have significantly advanced. Furthermore, with the recent discovery of novel therapeutic modalities for many inborn errors of metabolism, such as enzyme replacement therapies and substrate reduction therapies, the importance of newborn screening for these disorders is gaining more momentum. Especially that for most inborn errors, the earlier the specific therapy starts, the better the prognosis will be (Selim et al., 2014). Currently, hundreds of regional and national health care authorities all over the world have running programmes for the newborn screening of IEMs, which usually vary widely depending on the disease spectrum and financial limitations of each country.

Similar to the beginnings of NBS, metabolite-based screening is still the dominant form in all running programmes worldwide for the detection of various IEMs today. Almost two decades ago, tandem mass spectrometry (TMS) based techniques have revolutionized the metabolite screening for IEM allowing the multiplex screening of various metabolites and various diseases in the same run using the same sample with the added benefits of cost-effectiveness, high throughput, and low false positive and false negative rates (Pollitt et al., 1997; Rashed et al., 1997). This allowed the widespread use of TMS in the newborn screening for IEMs even in many developing countries in spite of their financial limitations (Hassan et al., 2016; Borrajo, 2021). However, the numbers of disorders that can be evaluated simultaneously by the same mass spectrometric methodology are limited. Furthermore, the sensitivity and specificity of the multiplex method toward various metabolites and the stability of these metabolites are not equal favoring the diagnostic outcomes of certain diseases over others.

Next generation sequencing in the form of whole exome and whole genome analysis is now being proposed strongly as a potential alternative for mass spectrometric newborn screening for IEMs (Narravula et al., 2017; Adhikari et al., 2020; Wojcik et al., 2021). These techniques have the advantages of high throughput, high accuracy and potentially being able to detect all types of genetic disorders even beyond the scope of inborn errors of metabolism with almost identical sensitivity and specificity. The transition to genetic diagnosis in newborn screening requires major logistical and ethical hurdles to be overcome. However, the major limiting factors include data interpretation dilemmas, which will be required to handle variants of uncertain significance in each individual, and the current relatively high cost of such techniques. Interestingly, with gaining experience through an actual newborn screening program, it is expected that variants of uncertain significance will be mostly reclassified into either benign/likely benign or pathogenic/likely pathogenic in the span of a few years. Furthermore, the running costs of genetic diagnostics are getting lower and lower by the day giving hope to the application of a universal screening technology for all hereditary disorders and leaving the role of metabolite analysis and biochemical assays for the second-tier and confirmatory tests. It seems like a second revolution in newborn screening for the detection of inborn errors of metabolism is approaching.

This Research Topic collection includes 13 articles from several countries applying newborn screening and genetic diagnosis for the detection of IEMs. Wang Y. et al. have discussed an important but commonly overlooked cause of sample contamination in newborn screening by TMS, which is the injection syringe. They summarized their experience in the Nanjing NBS programme in eastern China and the steps they performed to reach the actual cause of contamination. They further described how to treat and guard against the contamination recurrence in the future. Similarly, Peng et al. have provided valuable data about the important role the timing of sampling could play in impacting screening performances of mass spectrometric NBS programmes. They compared three sampling times: early (12–23 h), standard (24–48 h), and late (49–168 h) in a large population-based newborn cohort from California, USA. They found a significantly higher false positive rate for phenylketonuria testing when age at blood collection was between 12 and 23 h. Other analytes were also impacted in the early and late sampling groups.

Several studies included in this article collection have summarized the outcomes of different running NBS programmes in different countries. Maguolo et al. described their experience in the diagnosis, management and follow up of biotinidase deficiency patients showing key strategies and unsolved questions of the management of their patients in Verona, Italy. The study concluded that NBS introduction for biotinidase deficiency in Verona had a dramatic impact on its diagnosis and the incidence has increased significantly compared to other areas in Italy. Remec, Urh Groselj et al. described patients with very long-chain acyl-CoA dehydrogenase deficiency (VLCAD) diagnosed mainly through a recent expanded NBS programme by TMS that started in Slovenia in 2018. The study further investigated the genetic background of all screen positive newborns and reported four novel pathogenic variants in the *ACADVL* gene. Wang X. et al. reported on the diagnostic outcomes of their NBS programme of congenital adrenal hyperplasia in Nanjing, China over the period 2000–2019. During this period, over one million newborns were screened, and 62 patients were diagnosed with 21-hydroxylase deficiency with an incidence of 1/19,858 live birth. The study further reported 18 different pathogenic variants in the *CYP21A2* gene and commented on their genotype-phenotype correlations. A broader scope of the NBS programme for all detectable IEMs by TMS in Liuzhou, Southern China was reported by Tan et al. Hotspot mutations in *PAH* gene (Phenylketonuria), *IVD* gene (Isovaleric academia), *ACADS* gene (short chain Acyl-CoA dehydrogenase deficiency), *SLC22A5* gene (Creatine deficiency) and *GCDH* gene (Glutaric aciduria type-I) were reported.

Stinton et al. conducted a systematic review of test accuracy of acylcarnitines measurement in dried blood spots by TMS for the newborn screening of long-chain 3-hydroxyacyl-CoA dehydrogenase deficiency (LCHAD) and mitochondrial trifunctional protein deficiency (MTP). They showed that the positive predictive value of the test in the literature varied considerably, while sensitivity, specificity, and negative predictive value could not be calculated as the negatively screened newborns were not followed-up. Koracin et al. further evaluated the status of newborn screening for IEMs in South-Eastern-Europe through conducting detailed surveys about the services provided for the screening of IEMs in each of the 12 participating countries. Two consecutive surveys were conducted; the first was in 2013/2014, while the second was in 2020. The study concluded that the current status of NBS programs in South-Eastern-Europe is very variable and is still underdeveloped.

Pure genetic studies reporting novel phenotypes and novel genetic variants in families complaining of D-Bifunctional protein deficiency and sulfite oxidase deficiency were reported by Chen et al. and Zhao et al., respectively. Zeng et al. further evaluated the screening of *SLC25A13* genetic variants for the detection of citrin deficiency. In their study, a real-time PCR-based multicolor melting curve analysis was developed to detect the four most prevalent mutations in *SLC25A13* in the Chinese Han population in one closed-tube reaction. The melting curve analysis identified previously diagnosed patients accurately and determined the carrier frequency of the four common pathogenic variants in *SLC25A13* in 5,332 healthy newborns, which was surprisingly high.

Finally, the promise of replacing the metabolite-based newborn screening approach with the genetic-based approach has been extensively discussed in two reviews: Remec, Trebusak Podkrajsek et al. and Woerner et al. The first review focused on the technical feasibility, economic considerations, and clinical and ethical issues of the sequencing-based techniques, while the second review focused on the comparison between historical and current biochemical screening methods and current and future genetic methods of newborn screening, as well as the practical aspects and challenges for the use of genomic testing for NBS.

In conclusion, there is still a huge gap in most countries between the current applied technologies for the newborn screening of IEMs and the ideal situation, which gives every potentially diseased newborn the right to be diagnosed as early and as accurately as possible. We hope that with the application of the genetic NBS methods, we will be able to address the identified gap in the near future.

## Author Contributions

MAE drafted the manuscript that was reviewed and edited by LvH. Both authors co-edited the Research Topic.

## Conflict of Interest

The authors declare that the research was conducted in the absence of any commercial or financial relationships that could be construed as a potential conflict of interest.

## Publisher's Note

All claims expressed in this article are solely those of the authors and do not necessarily represent those of their affiliated organizations, or those of the publisher, the editors and the reviewers. Any product that may be evaluated in this article, or claim that may be made by its manufacturer, is not guaranteed or endorsed by the publisher.
